# Individual and Contextual Factors Associated With Malaria Among Children 6–59 Months in Burkina Faso

**DOI:** 10.3389/ijph.2023.1605347

**Published:** 2023-02-06

**Authors:** Ibrahima Stephane Sere, Aristide Romaric Bado

**Affiliations:** ^1^ Service De Neurologie, Centre Hopitalier Universitaire Sourou Sanou, Bobo-Dioulasso, Burkina Faso; ^2^ Research Institute for Health Sciences (IRSS), Ouagadougou, Burkina Faso; ^3^ West African Health Organisation (WAHO), Bobo Dioulasso, Burkina Faso

**Keywords:** child, malaria, determinants, multilevel regression, Burkina Faso

## Abstract

**Objective:** This study aims to understand the individual and contextual factors associated with malaria among children aged 6–59 months in Burkina Faso.

**Methods:** This cross-sectional study used secondary data extracted from the Burkina Faso Malaria Indicator Survey 2017–2018. Descriptive analysis was used to analyse socio-demographic characteristics. We performed a multilevel logistic regression model to highlight individual and contextual factors of children’s exposure to malaria.

**Results:** Our analysis included 5,822 children aged 6–59 months. Of these, 15% had a positive rapid diagnostic test. Factors associated with malaria among children 6–59 months were age, maternal education, household wealth, rural residence, and region. The variability in malaria exposure was 16% attributable to the strata level and 23% to the primary sampling unit level. Some factors, such as the family’s socio-economic status, access to hospital care, and place of living, were positively associated withs malaria cases in children.

**Conclusion:** The study identified some individual and contextual determinants of malaria among children aged 6–59 months in Burkina Faso. Taking them into account for the design and implementation of policies will undeniably help in the fight against malaria in Burkina Faso.

## Introduction

Malaria is a parasitic disease caused by a protozoan of the genus Plasmodium. “It is transmitted to humans by the bites of infected female *Anopheles* mosquitoes [1, 2]. Sub-Saharan Africa pays the heaviest price, with 95% of cases, 67% of which are in children under five years of age [3].

The World Health Organization’s (WHO) Global Malaria Report, released on 30 November 2020, highlighted an unprecedented period of success in the fight against malaria worldwide. Over the past two decades, malaria control had prevented 1.5 billion cases and 7.6 million deaths [3, 4]. On the one hand, progress in the fight against malaria has been achieved, through early diagnosis and appropriate case management. On the other hand, through vector control by the destruction of breeding sites, use of insecticide-treated mosquito nets, intra-domiciliary spraying of residual insecticide, and chemoprophylaxis (intermittent preventive treatment and seasonal chemotherapy) [5]. The National Malaria Control Programme (NMCP) has achieved satisfactory results up to 2015, reducing the mortality rate of children under five years of age from 129 to 81.6 per 1,000 inhabitants respectively from 2010 to 2015 [4, 6].

However, progress in the fight against malaria has stagnated in recent years in African countries with a high burden. The number of cases rose from 224 million in 2015 to 227 million in 2019 and 241 million in 2020. On the contrary’, the number of deaths declined from 736,000 in 2000 to 562,000 and 558,000 in 2015 and 2019, respectively. It then rose again to 627,000 in 2020, an increase of 69,000 more deaths than in 2019. Malaria deaths in sub-Saharan Africa increased by 12% in 2020 compared to 2019 [3].

Burkina Faso is one of the most affected countries. According to 2015 data from the Burkina Faso Ministry of Health, malaria was the leading reason for consultation (45.7%), hospitalisation (45.6%) and death (25.2%) in health facilities [4]. Burkina Faso is not on the side-lines, and the situation remains precarious, marked on the one hand by the weight of pre-existing difficulties, notably resistance to antimalarial drugs, the ineffectiveness of rapid diagnostic tests (RDTs) in the event of parasite mutation, the resistance of mosquitoes to insecticides, and the difficulties of financing and coordinating control actions. On the other hand, due to its geographical location, the country has been suffering since 2015 from the rise of terrorist attacks from neighbouring countries such as Mali and Niger, with the corollary of political instability and an unprecedented security, humanitarian, and health crisis.

The current instability in the country hinders the implementation and continuity of actions and policies to fight malaria. This situation is at the origin of migratory flows, population displacement, and a deterioration in living conditions, and affects the mobility of healthcare personnel. This environment causes an imbalance in malaria exposure particularly among children, and changes how this endemic is perceived.

Given the multiple challenges faced by the managers of NMCP, understanding the determinants of malaria based on factual data will enable the implementation of efficient health actions in line with the current challenges to achieve optimal control objectives. Malaria indicators are collected through the Malaria Indicator Survey (MIS), 2017. Previous MIS surveys were conducted in 2010 and 2014. In addition, a National Health Information System (NHIS) captures routine health service data regularly [7]. Considering the data provided by the 2017–2018 IPBES, our study aims to determine the individual and contextual factors associated with malaria in children aged 6–59 months in Burkina Faso.

We hypothesise that the high prevalence of malaria in children aged 6–59 months is the result of interrelated individual and contextual factors. In other words, do individual vulnerability characteristics influence the carriage of Plasmodium? Similarly, does the context of life (social vulnerability) and the household environment influence malaria infection in children aged 6–59 months?

The challenge of this study is to highlight the risk factors of malaria by bringing out the individual and contextual effects. Recognition of these factors will help health policy managers in their decision-making. This study has the following specific objectives: 1) to determine the socio-demographic characteristics of children under five years of age with a febrile condition in Burkina Faso, and 2) to identify the individual and contextual factors associated with malaria morbidity in children under five years of age with a febrile condition in Burkina Faso.

## Methods

### Study Area

**FIGURE 1:**
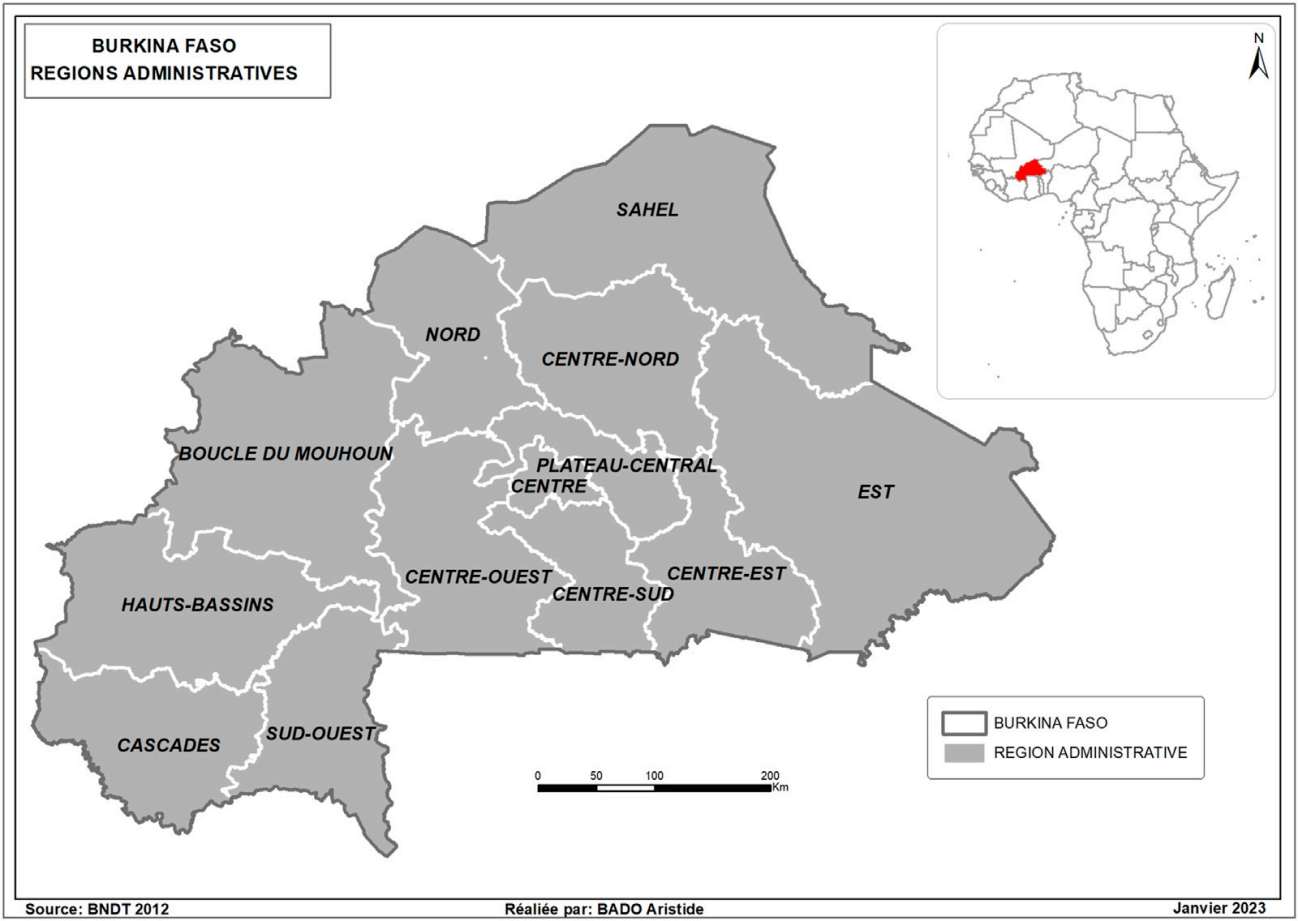
Map of Burkina Faso with administrative regions (Malaria Indicator Survey, Burkina Faso, 2017–2018).

Burkina Faso is located in sub-Sahara Africa with a superficies of 272,200 Km^2^ and is bordered to the north and west by Mali, to the northeast by Niger, to the southeast by Benin and to the south by Togo, Ghana, and Côte d’Ivoire. It ranks 185th out of 188 countries in the 2016 Human Development Index (HDI), published by the UNDP in 2017. The country’s population is characterised by its youth. The average age of the population was 16.6 years in 2006. Children under the age of 5 and 18 represented 17% and 53% of the population, respectively. It is divided into 13 administrative regions, characterised by cultural, socio-economic, and environmental diversity Figure 1.

### Source of Data

Data from the “Enquête sur les Indicateurs du Paludisme au Burkina Faso (EIPBF 2017–2018)” were used for this study. This is a nationally representative cross-sectional survey in which data were collected by the National Institute of Statistics and Demonography (*“Institut National de la Statistique et de la Démographie (INSD)”*) between December 2017 and March 2018 [6, 8]. The four databases set up were household (HR), household member (PR), mother (IR) and child (KR). The data from the mother (IR) and the child (KR) databases were aggregated for our study.

MIS aim to provide quality data to assess the progress of goals and targets necessary for effective monitoring and evaluation of NMCP meaurement implementation. Specifically, these surveys aim to assess insecticide-treated net (ITNs) ownership and use, coverage of the sporadic preventive care programme for pregnant women, and treatment-seeking behaviours. Additionally, it is an assessment of awareness, behaviours and behavioural indicators related to malaria control. Depending on the country’s needs, MIS can also identify the factors related to malaria and anaemia. In addition to these items, several other questions are asked about basic demographics and education.

### Sampling and Sample Size

A two-stage stratified cluster sample was used to determine the study sample. The primary sampling unit is the enumeration area (EA). The sampling was described in detail in the survey report (8). Each area was separated into urban and rural parts to form the sampling strata and the sample was drawn independently in each stratum. In total twenty-six sampling strata were created.

In the first stage, 252 EAs (52 urban, 200 rural) were drawn with probability proportional to size, where size is the number of households in the EA during the mapping exercise for the 2006 census.

In the second stage, from each of the EAs selected in the first stage, 26 households were selected (a total of 1,352 in urban areas and 5,500 in rural areas) that best represent the cultural and socio-economic diversity of the country as well as regional differences in malaria prevalence with a systematic equal probability draw from newly established lists at the time of enumeration. The sample size was calculated to provide statistically representative results on malaria prevalence in children aged 6–59 months [8, 9].

For this study, we examined 5,822 children aged 6–59 years who had a febrile episode during the two weeks preceding the survey and for whom the result of the malaria test (RDT) was available.

### Study Variables

#### Dependent Variable.

The response variable in our study is the result of the rapid malaria test (RDT) performed in children aged 6–59 months. The test result is coded “Positive” for a positive test for *Plasmodium falciparum* (Pf) and “Negative” if it is not.

Laboratory microscopy on blood smears and thickened drops was done for three-quarters of the households where RDTs were performed. Malaria results were also classified as positive or negative. There was a strong positive correlation of 0.581 (95% CI: 0.57–0.60; *p* < 0.0001) between these two test results. However, the laboratory microscopy test was performed primarily as a confirmation test for the RDT [9]. Thus, a malaria case was determined by a positive RDT with fever or a history of fever in the previous two weeks.

#### Independent Variables

The explanatory variables considered in our study were identified from the literature data [3, 7, 10–12]. The databases of children under five (KR) and household members (PR) were merged using a common primary key for both databases. The variables are divided into three groups, individual characteristics, household-level factors, and contextual factors [7, 9–14].

### Data Analysis

#### Descriptive analysis

A descriptive analysis using frequencies was used to establish the distribution of malaria status among children aged 6–59 months in Burkina Faso with the explanatory variables considered in our study. The explanatory variables were subjected to bivariate analyses to estimate the significance of their association with malaria (Table 2). The chi-square test with the second-order correction of Rao and Scott was used to compare proportions [15, 16].

#### Multivariate Analysis

Multilevel logistic regression was performed to identify individual and contextual effects. The hierarchical nature of the 2017–2018 EIPBF data easily allows the use of multilevel logistic regression models [17, 18]. Variables significant at the 20% level were retained for multilevel modelling. Despite non-significance at the 20% threshold, considering the data in the literature, some variables were retained for the following modelling [19].

Three multilevel logistic models were considered.

• Model 1: a null model or empty model, with no predictors. It is essentially a measure of the variation between strata (intra-stratum variance).
• Model 2: To model 1, the contextual variable EA or primary sampling unit was added. This was done to assess the variation between strata and EA (intergroup variance).
• Model 3: To model 2, individual variables were added.

The principle of parsimony was followed. A likelihood ratio test was performed to determine the most appropriate model [18, 20].

Measures of association (i.e., fixed effects) were described using an adjusted odds ratio (AOR) with corresponding *p*-values and 95% confidence intervals (CIs).

Measures of variation (i.e., random effects) were captured using the intra-class correlation (ICC). The ICC represents the proportion of the total variation in the dependent variable attributable to the contexts (strata or EAs). This coefficient will be 0 when there is no variance between the groups. In our model (three-level model), we identified two ICCs: one concerning children nested at the strata level and the groups at the strata level nested in the group at the administrative zone level. Therefore:

$${ICC}_{zad}=\frac{\sigma_{zd}^{2}+\sigma_{strate}^{2}}{\sigma_{zd}^{2}+\sigma_{strate}^{2}+\frac{\pi^{2}}{3}}$$ (1)

• ${ICC}_{zd}$ is the correlation between two children/individuals (unit of analysis) within the same stratum and administrative area.

$${ICC}_{menage}=\frac{\sigma_{strate}^{2}}{\sigma_{zd}^{2}+\sigma_{strate}^{2}+\frac{\pi^{2}}{3}}$$ (2)

• ${ICC}_{strate}$ is the correlation between two children/individuals in the same administrative area, but different in strata [17, 18].

From Eqs. 1, 2, the variance between EAs, is the variance between strata, and ≃ 3.29 is the variance between children/individuals with scale factor one for logistic regression [17, 18, 21].

The values of the CCIs help to establish the need for multilevel analysis versus single-level analysis. The rule of thumb could be: when the ICC is less than 5% in the null model, hierarchical modelling may not be necessary [22].

All analyses were performed using R software version 4.0.5. The “svydesign” command in the survey extension was used to adjust for under- and over-reporting in the survey, using a weighting factor of (v005/1000000), where v005 is the sample weight.

### Ethical Considerations

This study is based on the analysis of secondary data without the use of information about the identity of the participant. All DHS were approved by ICF International and a national ethics committee in each host country. All participants gave written informed consent before taking part in the survey. Although, additional ethical approval was not required in this study, we obtained written permission from the DHS programme to use the data.

## Results

### Characteristics of the Study Population

Table 1 presents a description of the study variables. One in five children was febrile during the survey and 15% of RDTs were positive with 3% cases of anaemia.

**TABLE 1:** Description of study variables (Malaria Indicator Survey, Burkina Faso, 2017–2018).

Variables	Number (n)	Percentage (%)
Result of the rapid diagnostic test for malaria		
Negative	4,970	85.4
Positive	852	14.6
Age of the children		
6–11 months	1,321	22.7
1–2 years	1,091	18.8
2–3 years	1,118	19.2
3–4 years	1,177	20.2
4–5 years	1,115	19.1
Gender of children		
Female	2,846	48.9
Male	2,976	51.1
Anaemia in children		
Presence of anaemia	184	3.16
Absence of anaemia	5,638	96.84
The febrile status		
Feverish	1,182	20.3
Non-febrile	4,640	79.7
Age of mother		
15–24 years	1,532	26.3
25–34 years old	2,781	47.8
35 years and over	1,509	25.9
Level of education		
Not in school	4,445	76.3
Primary	806	13.9
Secondary	536	9.2
Superior	35	0.6
Socio-economic status of the household		
Poor	2,474	42.5
Medium	1,181	20.3
Rich	2,167	37.2
Possession of a mosquito net		
No	1,131	19.4
Yes	4,691	80.6
Use of mosquito netting		
No	3,362	57.8
Yes	2,460	42.2
Effective use of care		
No	4,948	85.0
Yes	874	15.0
Place of recourse		
None/self-medication/private/tradipractitioner	4,988	85.7
Public sector	834	14.3
Quality of care		
Suitable	747	12.8
Not suitable	5,075	87.2
Contextual variables		
Primary sampling unit		
Place of residence		
Urban	930	16.0
Rural	4,892	84.0
Region		
Boucle du Mouhoun	615	10.6
Cascades	271	4.7
Centre	348	6.0
Central East	477	8.2
North Central	531	9.1
Central West	596	10.3
South Central	173	3.0
East	628	10.8
Hauts-Basins	752	12.9
North	501	8.6
Plateau Central	294	5.0
Sahel	414	7.1
South-West	222	3.8
N	5,823	100

At the household level, the mothers were young, with three-quarters (74%) being under the age of 34. The majority of mother had no school education (76%). About two-thirds of the children were from households with low socio-economic status (62%). Four-fifths of households owned ITNs (81%) obtained from mass distribution campaigns or by themselves. However, there is low usage of this control method. Only one in two children slept under a net the day before the survey. In terms of context, most children live in rural areas (84%). The Hauts-Bassins (13%), the Boucle du Mouhoun (11%), and the East (11%) are the most represented regions. Conversely, the least represented were Cascades (4.7%), South-West (3.8%), and Centre-Sud (3%).

### Bivariate Analysis

Table 2 shows that child age, presence of anaemia, mother’s education, household socio-economic status, ITN use, residence and region are significantly associated with malaria in children (*p* < 0.05). Thus, there are fewer cases of malaria in infants aged 6–11 months (5.7%) compared to older children, 3–4 years (21%), and 4–5 years (20%). Also, one-third of malaria cases were severe forms related to anaemia (33%). Children with young mothers (16%) and from poor households (16%) were more affected. Finally, most malaria cases reside in rural areas. The most affected regions were the South-West, the Boucle du Mouhoun and the Centre-West.

**TABLE 2:** Cross-tabulation of malaria status with child, parent, community, and administrative area predictors (Malaria Indicator Survey, Burkina Faso, 2017–2018).

Variables	RDT results		p-value
	Negative	Positive	
	n (%)	n (%)	
Individual level			
Socio-demographic characteristics of children			
Age of the children			<0.001
6–11 months	1,246 (94.0)	75 (5.7)	
1–2 years	947 (87.0)	145 (13.0)	
2–3 years	946 (85.0)	171 (15.0)	
3–4 years	934 (79.0)	243 (21.0)	
4–5 years	897 (80.0)	218 (20.0)	
Gender of children			0.8
Female	2,433 (85.0)	414 (15.0)	
Male	2,537 (85.0)	439 (15.0)	
Clinical characteristics of children			
Anaemia in children			<0.001
Presence of anaemia	123 (67.0)	61 (33.0)	
Absence of anaemia	4,846 (86.0)	792 (14.0)	
The febrile status			0.3
Feverish	995 (84.0)	187 (16.0)	
Non-febrile	3,975 (86.0)	666 (14.0)	
Socio-demographic characteristics of mothers			
Age of mother			0.4
15–24 years	1,311 (85.0)	223 (15.0)	
25–34 years old	2,392 (86.0)	389 (14.0)	
35 years and over	1,267 (84.0)	241 (16.0)	
Level of education			<0.001
Not in school	3,729 (84.0)	715 (16.0)	
Primary	712 (88.0)	95 (12.0)	
Secondary	493 (92.0)	43 (8.0)	
Superior	35 (100.0)	0 (0.0)	
Socio-economic status of the household			0.0153
Poor	2,067 (84.0)	407 (16.0)	
Medium	1,002 (85.0)	179 (15.0)	
Rich	1,901 (88.0)	266 (12.0)	
Means of combating malaria			
Possession of a mosquito net			0.11
No	943 (83.0)	188 (17.0)	
Yes	4,027 (86.0)	664 (14.0)	
Use of mosquito netting			0.015
No	2,912 (87.0)	450 (13.0)	
Yes	2,058 (84.0)	402 (16.0)	
Use of care			
Effective use of care			0.12
No	4,207 (85.0)	742 (15.0)	
Yes	762 (87.0)	110 (13.0)	
Place of recourse			0.062
None/self-medication/private/tradipractor	4,238 (85.0)	750 (15.0)	
Public sector	731 (88.0)	103 (12.0)	
Quality of care			0.9
Suitable	638 (85.0)	109 (15.0)	
Not suitable	4,331 (85.0)	744 (15.0)	
Contextual variables			
Place of residence			0.001
Urban	890 (96.7)	40 (4.3)	
Rural	4,080 (83.0)	812 (17.0)	
Region			<0.001
Boucle du Mouhoun	491 (80.0)	124 (20.0)	
Cascades	238 (88.0)	33 (12.0)	
Centre	326 (94.3)	20 (5.7)	
Central East	424 (89.0)	54 (11.0)	
North Central	449 (85.0)	82 (15.0)	
Central West	461 (77.0)	136 (23.0)	
South Central	154 (89.0)	19 (11.0)	
East	532 (85.0)	97 (15.0)	
Hauts-Basins	665 (88.0)	87 (12.0)	
North	456 (91.0)	45 (9.0)	
Plateau Central	272 (93.0)	22 (7.5)	
Sahel	353 (85.0)	62 (15.0)	
South-West	150 (67.0)	72 (33.0)	

### Multilevel Analyses

Table 3 presents the results of the multilevel logistic models of malaria prevalence (Quick Test). The adjusted odds ratios (AOR) for each of the variables considered in the analysis after adjustment for the other variables, showed that the variables were significantly associated with the occurrence of malaria.

**TABLE 3:** Multilevel logistic regression results for factors associated with malaria risk in children aged 6–59 months (Malaria Indicator Survey, Burkina Faso, 2017–2018).

Variables	Model 1		Model 2		Model 3	
	AOR	95% CI	AOR	95%CI	AOR	95%CI
Individual level						
Age of the children						
6–11 months					1	
1–2 years					2.83***	2.09–3.81
2–3 years					3.4***	2.53–4.58
3–4 years					4.75***	3.57–6.33
4–5 years					4.13***	3.08–5.55
Anaemia in children						
Absence of anaemia					1	
Presence of anaemia					3.14***	2.18–4.52
The febrile status					1	
Feverish					1.89***	1,39–2,56
Non-febrile						
Level of education of mothers						
Not in school					1	
Primary					0,84	0.65–1.08
Secondary and above					0.59***	0.41–0.85
Age of mothers						
15–24 years					1	
25–34 years					0.87	0.58–1.29
35–49 years old					0.82	0.50–1.34
Possession of a mosquito net						
No					1	
Yes					1.01	0.79–1.27
Use of mosquito netting						
No					1	
Yes					1.08	0.88–1.31
Socio-economic status of the household						
Poor					1	
Medium					0.9	0.7–1.15
Rich					0.59	0.38–0.85
Effective use of care						
No					1	
Yes					0.5	0.35–0.71
Region of residence						
Centre					1	
Boucle du Mouhoun					1.43	0.68–3.01
Cascades					1.08	0.51–2.30
Centre est					0.81	0.37–1.73
Centre nord					1.23	0.58–2.60
Centre ouest					2.12***	1.01–4.45
Centre sud					0.78	0.35–1.75
Est					1.06	0.50–2.23
Hauts-bassins					1.06	0.50–2.27
Nord					0.62	0.29–1.34
Plateau central					0.5	0.23–1.11
Sahel					1.16	0.52–2.61
Sud-Ouest					3.83***	1.83–8.02
Place of residence						
Urban					1	
Rural					2.46	1.6–3.8
Random effects						
a σ^2^ (π^2^/3)					3.29	
Variance of random intercept					0.38	
					0.46	
Conditional intraclass correlation					0.2	
Log likelihood	−2,373.79		−2,333.91		−2,192.778	
AIC	4,751.58		4,673.82		4,447.91	
Number at first level units (Strates)	24		24		24	
Number at second level units	245		245		245	

*p ≤ 0.1, **p ≤ 0.05, and ***p ≤ 0.01, AOR, Ajusted Odds Ratio; 95% CI, Confidence Interval.

^a^ Assumed standard logistic normal distribution for individual level variances (2/3).

#### Fixed Effects Analysis

Model 3 shows that the variables age of the child, presence of anaemia, the existence of febrile symptoms, level of education of the mothers, household wealth index, early use of healthcare, and region and area of residence are significantly associated with exposure to malaria among children aged 6–59 months in Burkina Faso.

The model shows that children’s exposure to malaria increases with age. Thus, the AOR increases from 2.83 (CI = [2.09–3.81], *p* < 0.001) for infants aged 1–2 years to 4.75 (CI = [3.57–6.33], *p* < 0.001) for children aged 3–4 years. Furthermore, this risk was aggravated by anaemia and fever, with an AOR of 3.14 (CI = [2.18–4.52], *p* < 0.001) and 1.89 (CI = [1.39–2.56], *p* < 0.001) respectively. In addition, region and residence favoured malarial exposure with AORs of 3.83 (CI = [1.83–8.08], *p* < 0.001) for the south-west region and 2.41 (CI = [1.60–3.80], *p* < 0.001) for rural residents. However, the mothers’ education level, high wealth index, and early recourse to care would be the protective factors. Indeed, reaching the secondary school level would reduce the risk of malaria by 41% (OR = 0.59; CI = [0.41–0.85]; *p* < 0.005), belonging to wealthy households would reduce exposure by 43% (OR = 0.59; CI = [0.38–0.85]; *p* < 0.006) while seeking care reduced the risk by 50% (OR = 0.50; CI = [0.35–0.71]; *p* < 0.001).

#### Analysis of Variable Effects

The stratum and primary sampling unit factors contributed 39% and 62% of the variance in the null model (without covariates) respectively. The inclusion of household covariates reduced this variance component to 38% and 46%, respectively, suggesting that these variables explain about 16% of the inter-household variability in the probability of malaria entry.

## Discussion

Our study sought to highlight the individual and contextual factors associated with malaria in children aged 6–59 months in Burkina Faso. Firstly, the proportion of malaria cases was estimated at 15%, with regional inequalities. This result is lower than that of previous PIA-BFs, with 46% in 2014% and 66% in 2010 [23]. It is also lower than those of Obasohan et al. who found that 35.5% of children had malaria in Nigeria (DHS 2018) [10]. This low proportion is thought to be due to the timing of data collection. The 2010 and 2014 PIT-BF data were collected from May 2010 to January 2011 and October to November 2014. The data collection for our study took place from November 2017 to March 2018. In addition, for 91% of children, malaria data were collected during January, February, and March. This period is completely outside the high malaria transmission season in Burkina Faso, which runs from May to October [8]. This time lag would lead to an underestimation of the malaria burden in children.

Secondly, the study investigated the vulnerability profiles to malaria, which show that although the risk exists everywhere, its distribution is uneven, with a greater risk for populations living in rural areas, particularly in the south-west region. Exposure to malaria depends on factors, such as the location of the vector (female *Anopheles*), the intermediate host (humans), and the parasite and the environment. Rural areas and the southwestern region are areas of intense and permanent transmission due to climatic conditions characterised by high rainfall and a high concentration of breeding sites that favour the multiplication of mosquitoes (a long-life span that allows the parasite to complete its evolutionary cycle). In addition, the vulnerability of rural populations is accentuated by a lack of health centres compared to urban areas, which reduces the access to care.

Finally, considering the correlation of individual and contextual factors, the nested nature of our data, allowed us to avoid the ecological error that would result from their dispensation. Thus, after adjusting for all variables, the results of the study highlight the socio-environmental factors related to malaria exposure. Exposure to malaria increases significantly with age [24], fever and anaemia, lack of or low level of education of mothers [25] and a low household wealth index [25]. Our results are consistent with the literature [10, 26].

Thus, older children are at greater risk of malaria. Musuyi [23] in the Democratic Republic of Congo and Obasohan [10] in Nigeria have made the same finding. This could be explained by the fact that infants have maternal antibodies received *in utero* and this protection against infection is reinforced by breastfeeding. While older infants are in ablactation and have not yet developed immunity to malaria [27]. In addition, infants receive more care from mothers and nannies. They sleep under mosquito nets with their mothers and are dressed to cover their whole bodies, which makes them less susceptible to mosquito bites. While the older children are more mobile and receive less care and attention than their younger siblings, they also sleep less under nets and will be more exposed to mosquito bites. Our results are consistent with those of Tassembedo et al. [28] in Burkina in 2018, Masui [23] and Obasohan [10].

Furthermore, our results show an association between cases of fever, anaemia, and exposure to malaria. These are two consequences of malaria that represent signs of disease severity [3, 4, 29]. The study by White et al. showed the negative impact of malaria on the occurrence of anaemia [30]. Plasmodium leads to the destruction of red blood cells with a consequent decrease in haemoglobin levels. Stark et al. found in a longitudinal study in Burkina Faso in 2021 that anaemia persisted after the negativation of sputum drops and smears in children [7]. Delay or lack of adequate management will result in severe anaemia leading to severe malaria. Mukisa et al. [27] in Uganda have established a similar link between malaria and anaemia.

In addition, children whose mothers are not in school or have a low level of education and those from households with a low wealth index are more exposed than children whose mothers have a higher level of education and come from wealthier households. Mothers’ education modulates how they perceive malaria risk and influences their knowledge, attitudes, and practice of malaria prevention. Educated mothers better assimilate prevention messages and apply them. Also, educated mothers come from wealthy households. They live in healthy environments. These households have the financial means to equip their homes with mosquito-proofing devices, such as screens on doors and windows, insecticide-treated mosquito nets and repellents. All these factors reduce contact with mosquitoes and thus exposure to the risk of malaria. Our results are similar to those of Tassembedo [28], Samadoulougou [14], Musuyi [23] and Obasoyan [10].

The results of the study show that early access to care significantly reduces exposure to malaria. Early detection and appropriate management reduce morbidity. This will lead to a reduction in infected mosquitoes. All of which will reduce exposure to malaria. However, this is often delayed, and mothers often resort to self-medication before going to a health centre. The results of a study in Burkina Faso showed that 72.7% of cases treated for suspected malaria in a district hospital had self-medicated before admission [31]. Similar results were found by other studies highlighting the use of self-medication before seeking appropriate care [32–35].

Our study was made possible by the availability of DHS data. It uses a rigorous and reproducible sampling and collection methodology covering the entire territory and guaranteeing the reliability of the data. However, this study has some limitations. On the one hand, due to the cross-sectional nature of the data collected, the explanatory variables and the dependent variable were measured at the same time. It is therefore not possible to guarantee any causality of the associations. On the other hand, the collected information based on the respondents’ memories could be a cause of memory bias. Disaggregated studies should be considered to determine the factors at the individual level, considering the environmental factors as well.

### Conclusion

The results of this study highlighted the socio-spatial inequalities associated with the risk of malaria in children aged 6–59 months. The study found that the age of the child, presence of anaemia, the existence of febrile symptoms, level of education of the mothers, household wealth index, early use of healthcare, region and area of residence are individual-level factors significantly associated with exposure to malaria among children aged 6–59 months in Burkina Faso. The study also highlighted the effect of contextual factors. Indeed, the variability in malaria exposure was 16% attributable to the strata level and 23% to the primary sampling unit level. It is important to take these factors into account for monitoring and evaluating the NMCP e and for raising awareness strategies for malaria control. The results of this study can contribute in reducing the incidence of malaria and preventing its resurgence.
